# Identification and Candidate Gene Evaluation of a Large Fast Neutron-Induced Deletion Associated with a High-Oil Phenotype in Soybean Seeds

**DOI:** 10.3390/genes15070892

**Published:** 2024-07-08

**Authors:** William R. Serson, Mohammad Fazel Soltani Gishini, Robert M. Stupar, Adrian O. Stec, Paul R. Armstrong, David Hildebrand

**Affiliations:** 1 Department of Biology, Penn State University, Lehigh Valley, Center Valley, PA 18034, USA; 2 Department of Plant Pathology, University of Kentucky, Lexington, KY 40546, USA; mohammad.soltani@uky.edu; 3 Department of Agronomy and Plant Genetics, University of Minnesota, Saint Paul, MN 55108, USA; stup0004@umn.edu (R.M.S.); stecx002@umn.edu (A.O.S.);; 4 United States Department of Agriculture-Agricultural Research Service, Manhattan, KS 66502, USA; 5 Department of Plant and Soil Sciences, University of Kentucky, Lexington, KY 40546, USA; dhild@uky.edu

**Keywords:** fast neutron mutagenesis, renewable oil, triacylglyceride, comparative genomics hybridization

## Abstract

Since the dawn of agriculture, crops have been genetically altered for desirable characteristics. This has included the selection of natural and induced mutants. Increasing the production of plant oils such as soybean (*Glycine max*) oil as a renewable resource for food and fuel is valuable. Successful breeding for higher oil levels in soybeans, however, usually results in reduced seed protein. A soybean fast neutron population was screened for oil content, and three high oil mutants with minimal reductions in protein levels were found. Three backcross F2 populations derived from these mutants exhibited segregation for seed oil content. DNA was pooled from the high-oil and normal-oil plants within each population and assessed by comparative genomic hybridization. A deletion encompassing 20 gene models on chromosome 14 was found to co-segregate with the high-oil trait in two of the three populations. Eighteen genes in the deleted region have known functions that appear unrelated to oil biosynthesis and accumulation pathways, while one of the unknown genes (*Glyma.14G101900*) may contribute to the regulation of lipid droplet formation. This high-oil trait can facilitate the breeding of high-oil soybeans without protein reduction, resulting in higher meal protein levels.

## 1. Introduction

There is a strong genetic component that is well understood for oil content and quality via the oil biosynthetic pathway and its regulation. Seed oil is biosynthesized during the second main stage of seed maturation [1,2,3], at which time the relevant biosynthetic enzymes are highly expressed. For instance, in studies of expression profiles of triacylglyceride (TAG) biosynthetic enzymes and oil accumulation in developing soybeans (*Glycine max*), *DGAT1* shows an expression profile suggesting a dominant role in soybean oil biosynthesis, but *DGAT2* and *PDAT* do not [4,5,6].

It is becoming increasingly possible to alter hydrocarbon flux in soybeans. Multiple studies indicate that oil content increases with a higher expression of TAG biosynthetic genes [7,8,9]. Increased expression of regulatory genes that upregulate multiple enzymes for fatty acid biosynthesis also can result in higher oil levels [9]. Co-expression of the transcription factor *WRI1* with *DGAT1*, a key rate-limiting enzyme, is shown to have a synergistic effect on TAG biosynthesis in plants [10,11,12]. Overall, increasing sink strength results in increased oil and protein, with a strong pronounced effect on protein and less on oils [13].

Cultivar effects are well understood to affect protein content and amino acids in soybeans, most likely due to heritable differences in TAG biosynthetic genes and regulatory factors. For instance, germplasm line N6202 produced seeds with 45.7% protein content and a 10% reduction in grain yield compared to a control variety, NC-Roy [14]. In contrast, TN03-350 and TN04-5321 achieved 43.1–43.9% protein content without sacrificing seed yield. Considering the importance of grain yields in commercial varieties, such a result is more desirable than a decrease in yield with improved grain quality. In soybean genotypes of early maturity groups, average-to-high protein content (399–476 g/kg^−1^) was found in years with high air temperature and moderate rates of rainfall during the seed-filling period, whereas seed protein content was drastically reduced (265–347 g/kg^−1^) in seasons of insufficient nitrogen fixation or higher amounts of precipitation during seed filling [15].

In plant breeding, random mutagenesis is a common way to generate mutations and increase genetic diversity for traits with limited natural variation. Common examples include the use of a chemical mutagen like ethyl methane sulfonate (EMS) or bombardment with gamma radiation, and today even site-directed mutagenesis is possible [16]. While these methods can produce useful traits, such as an early flowering mutant or increased oil content, it is possible that other important genes could be mutated, causing undesirable phenotypes, such as altered seed composition. However, mutagenized populations are not brought under the same scrutiny as transgenic approaches, and therefore traits induced this way are much easier to incorporate into the existing germplasm.

While utilization of mutagens can introduce much-needed levels of genetic and phenotypic diversity, it is imperative to understand the nature of the mutations induced. The mutagen used in this study, fast neutron bombardment (FN), typically induces deletion and/or chromosomal rearrangement events from several base pairs-long to several megabases [17,18,19]. The phenotypic variation can be used to study the association of genes with specific traits [20] or as a source of new variation for breeding purposes [21,22,23]. Comparative genomic hybridization (CGH) is one of the fastest and most effective ways to assess duplications or deletions caused by irradiation mutagenesis, such as FN. This technology utilizes oligonucleotide probes affixed to slides, known as microarrays. Fluorescently labeled DNA samples can then be hybridized to the probes, thus emitting a fluorescence intensity proportional to the DNA sample copy number for each probe sequence. The mutant DNA sample can be compared to a control sample (the non-mutagenized parent DNA) to identify tracts of sequence that have been duplicated or deleted in the mutant compared to its parent line [17,18,24]. In the case of the current study, the soybean microarray consists of 700,000+ features, allowing for a probe spacing of approximately 1000 base pairs across the euchromatic portion of the genome. With the advances of next generation sequencing and microarray technology, combined with intimate knowledge of the soybean genome, we can now harness CGH technology to quickly and precisely assess copy number variants (CNVs) in segregating mutant backcrosses.

Here, we utilized a fast neutron population of soybeans which exhibit 3-to-4% higher oil content than the parent variety with only a minor decrease in protein, resulting in seeds with increased oil plus protein content compared to the parent varieties. We hypothesize that the loss of a specific gene or genes within the deleted 300 kb region on chromosome 14 is responsible for the high-oil phenotype. Our analysis provides new insight into which of these genes may be the most likely to cause this change and may be the best candidate for future functional analyses.

## 2. Materials and Methods

### 2.1. Genetic Material

Seeds from three mutant lines, 1R22C28Cgadbr355aMN13, 5R12C21Dar387dMN13, and 5R16C01Dar388eMN13, and their parent, M92-220, from the University of Minnesota, were requested from their fast neutron (FN) mutant library in April 2014 and planted in the field in Lexington, KY, during the summer of 2014 to verify high-oil content in that environment using a single-line, randomized complete block design with border seed of the “Jack” cultivar. Ten plants were in each line, with three total replications, with each plant individually bulked and analyzed via non-destructive NIRS [25,26,27].

M92-220 is a maturity group “I” soybean produced by the University of Minnesota soybean breeding program. It is derived from the cultivar “MN1302” (PI 616498), which was originally selected from a cross between “Hendricks” × “Archer” [28]. M92-220 exhibits an indeterminate growth habit, purple flowers, grey pubescence, brown pods, yellow seeds, and a buff hilum.

### 2.2. Backcrossing

Two mutant lines (1R22C28Cgadbr355aMN13 and 5R12C21Dar387dMN13) in the M8 and M5 generations, respectively, exhibited high oil content in the KY environment and were thus planted and backcrossed to the parent line M92-220 in 2015, similar to other methodologies [18]. Successful crosses were harvested, and eleven F1 crosses were assessed for oil content via single-seed NMR. These were then grown in a greenhouse in the winter of 2015–2016 and in the spring of 2016. Thirty F2 seeds from each of the F1 plants were assessed for single-seed oil content and then planted at Spindletop Farm in Lexington, KY. In addition, 30 other randomly selected seeds were planted for each line, bringing the total to 60 total F2 seeds from F1 plants planted for each line.

### 2.3. Tissue Sampling, Seed Composition, and DNA Extraction 

Leaf tissue was collected and frozen at −80 °C [29] for comparative genomic hybridization (CGH) analysis from F2 plants. Seeds were harvested from each plant in the fall, and each F2 plant’s F3 seed was analyzed in bulk with a Perten DA7200 NIRS for oil, protein, and moisture content. These data were used to generate scatter plots of the F2 sibs. Strong inverse correlations of oil and protein content suggested that segregations of the mutant trait were found in three F2 populations.

### 2.4. NMR Methods

Oil content was determined by single-seed NMR, using a Minspec 20 (Bruker Biospin, The Woodlands, TX, USA) [25]. The instrument accommodates a 20 mm sampling-tube diameter. Seeds were weighed and placed into the tube and allowed to warm to 40 °C before insertion into the instrument. The standard oil seed measurement procedure supplied with the instrument controller was used. Calibration of the NMR instrument was performed using weighed amounts of extracted soybean oil encompassing the range of oil weights of the seed samples. Four different weights were used and were expressed on tissue paper at the bottom of the 20 mm sample tube.

### 2.5. CGH Analysis

DNA was extracted on a per-plant basis using a QIAGEN Plant DNeasy Kit from the leaves of three F2 populations (known here as A1, A2, and A3) expected to segregate for seed-oil content. The F2 plants that gave rise to F2:3 seeds with the highest seed oil (“high-oil” plants) and lowest seed oil (“normal-oil” plants) were respectively identified using NIRS. The DNA from the high-oil F2 plants was bulked, and the DNA for the normal-oil F2 plants was bulked for each of the three populations. The bulked DNAs were then subjected to CGH analyses, as previously described [17], using a custom NimbleGen CGH microarray with over approximately 700,000 probes, approximately 1 probe every 1 kb [17]. The CGH probe positions were designed according to the soybean cultivar Williams 82 genome version 2 assembly (Wm82.a2.v1). Each CGH was performed as a comparison within each population (e.g., A1 high-oil versus A1 normal-oil, etc.) From these comparisons, a large deletion was detected on chromosome 14, and the genes in that region (according to the Williams 82 version Wm82.a2.v1 gene annotation set) were analyzed on soybase.org and cross-referenced to homologous genes in the TAIR database.

### 2.6. Functional Analysis

SignalP-6.0 (https://services.healthtech.dtu.dk/services/SignalP-6.0/, accessed on 1 January 2024) was used to investigate possible signal peptides. The topology analysis was performed by Protter (https://wlab.ethz.ch/protter/start/, accessed on 1 January 2024) [30], and the protein network was established using the STRING database (https://string-db.org, accessed on 1 January 2024) [31]. Interactions in STRING are derived from genomic-context predictions, high-throughput lab experiments, co-expression, automated text-mining, and previous knowledge in databases. Phyre2, also known as Protein Fold Recognition Server, is a web portal for protein modeling, prediction, and analysis (http://www.sbg.bio.ic.ac.uk, accessed on 1 January 2024) [32]. Phyre2 was used to study the function of unknown genes in this study, and Chimera software version 1.11.2 was used to visualize the structural protein models [33].

## 3. Results

### 3.1. Oil and Protein Content of Backcrosses

Three mutant lines were selected for analysis based on previously observed high seed-oil content when grown in MN. The full names of these lines (1R22C28Cgadbr355aMN13, 5R12C21Dar387dMN13, and 5R16C01Dar388eMN13) are abbreviated herein as “1R22”, “5R12”, and “5R16” for simplicity. When grown in KY in 2015, these lines again showed a higher mean seed oil content, though not as pronounced compared to their wild-type parent line, M92-220, as was previously observed in MN (Table 1). We attempted to backcross the mutants to M92-220, resulting in eleven successful crosses. However, successful crosses were only observed for two of the mutants, 1R22 and 5R12.

**Figure 1:**
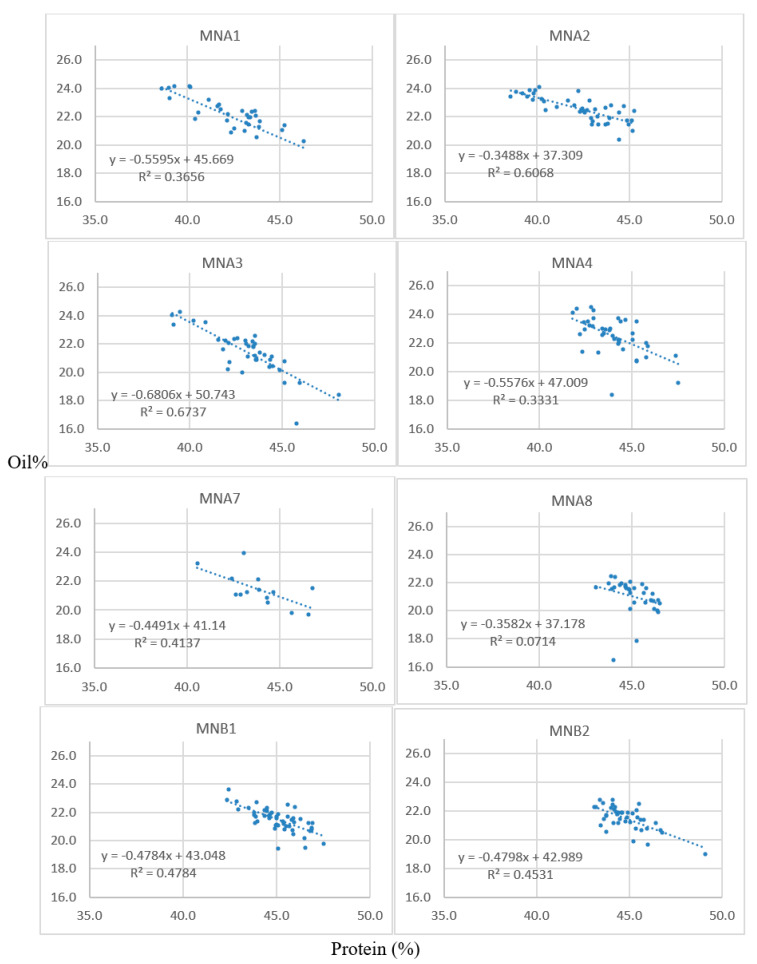
Scatter plots of the protein (*x*-axes) and oil (*y*-axes) content of F2:3 seeds. Each data point represents the NIRS values for each respective F2:3 family (e.g., corresponding to the rows in Table 5 for the A1, A2, and A3 populations). Seeds were analyzed for percent oil and protein content on a dry-weight basis via bulk-seed NIRS with n = 3 technical replications for each F2:3 seed lot.

Successful crosses were obtained between 1R22 × M92-220 and 5R12 × M92-220. The oil content of the resulting F1 seeds ranged from 17.3 to 22.5% (Table 2), as was determined by single-seed NMR. These F1 seeds were grown in a greenhouse over winter and designated as A1 through B3 plants. A5, A6, A7, and B3 seeds were planted but were not viable or did not survive to maturity. The F2 seeds resulting from the greenhouse planting were harvested and bulked to measure oil and protein content for all the seeds from each F2 population via NIRS (Table 3). Furthermore, thirty seeds were randomly selected from each of these populations for single-seed NMR analysis. Table 4 shows data from the A1 population. These 30 F2 seeds analyzed via NMR were then planted in the field for each respective population, along with an additional 30 F2 seeds that did not contain single-seed data per population. Leaf tissue was collected during the two-leaf stage for the F2 plants, and the F3 seeds were harvested at maturity. NIRS bulk data were collected on each F2:3 sample, using approximately 100 seeds. The protein and oil estimates for these families are shown for lines derived from the A1, A2, and A3 F1 lineages, which are all from crosses between 1R22 and the M92-220 WT (Table 5). Scatter plots of the protein and oil estimates for a deeper sampling of F2:3 families are shown (Figure 1); each data point represents the values for a given F2:3 sample of seeds. The populations generally showed good spread in their data, with inverse correlations between protein and oil. This indicates that a genetic factor may have segregated in the F2 plants, perhaps an FN-induced mutation that is associated with the high-oil trait.

### 3.2. CGH Analysis Reveals a Strong Candidate Deletion for the High-Oil Mutant Phenotype

We chose to further investigate the A1, A2, and A3 populations to determine if an FN-induced deletion was co-segregating with the high-seed-oil phenotype in the 1R22 mutant populations. We collected DNA for each of the F2 plants grown in the field in KY. We performed a bulk segregant analysis using CGH to see if there were any deletions that co-segregated with the high-oil phenotype. Based on the F2:3 NIRS data (Table 5), we binned each F2 into those that gave rise to high seed oil (high-oil, bold) and those that gave rise to normal seed oil levels (normal-oil, non-bold) within each respective population. We then bulked the DNA for the high-oil and normal-oil plants, respectively. These bulked DNA samples were subjected to CGH analyses to compare the deletion/duplication profile of the high-oil and normal-oil bulks for each of the three populations. The deletion/duplication profile of the high-oil and normal-oil bulks for each of the three populations was determined. We expected that the CGH profile would identify a differential hybridization between any deletion that was enriched in the high-oil segregating bulk of individuals compared to the normal-oil segregating bulk.

**Figure 2:**
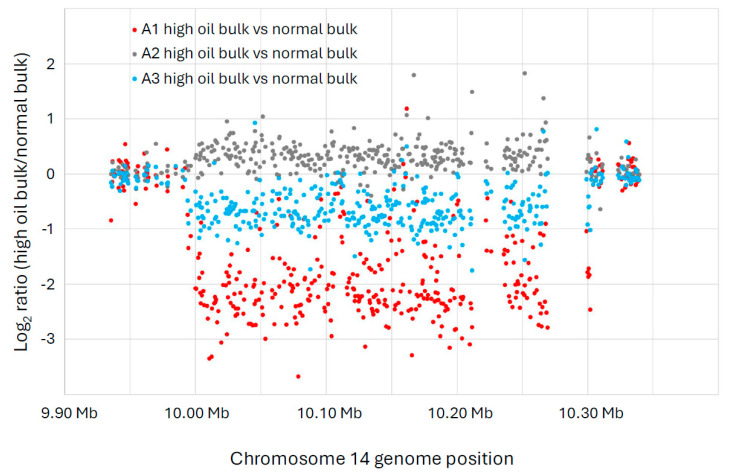
A large copy number variation (CNV) event detected in chromosome 14 of the A1 and A3 populations (both derived from separate crosses between 1R22 × WT) exhibited strong inverse correlations of oil and protein content. The *x*-axis indicates the location of each microarray feature along chromosome 14, according to the Williams 82 genome version 2 assembly (Wm82.a2.v1). The *y*-axis shows the log2 ratio of the CGH intensity from the high-oil versus the normal-oil bulk for each microarray feature. The blue dots show the CGH comparison between A1 high-oil versus A1 normal-oil. The orange dots show the CGH of high-oil versus normal-oil for A2, and the grey dots show the CGH of high-oil versus normal-oil for A3. These data indicate that a large deletion is enriched in the high-oil plants of the A1 and A3 populations, while the A2 population does not show this enrichment. This deletion event was detected on Chr14, from bp 9,994,086 to 10,301,954, a span of approximately 308 kb.

One large deletion event (>300 kb) on chromosome 14 was enriched in the high-oil bulks of the A1 and A3 populations (Figure 2), as evidenced by the string of data points below the log2 value of zero. (Zero indicates no difference between the high-oil bulk and the normal bulk, whereas values below zero indicate an enrichment for an FN deletion in the high-oil bulks compared to the normal bulks.) Specifically, the deletion was found on Chr14, from bp 9,994,086 to 10,301,954, a span of approximately 308 kb. The deletion is more pronounced in the A1 population, indicating that a greater proportion of individuals in that population were likely homozygous for the large deletion. Nonetheless, the A3 population also shows an enrichment for the large deletion in the high-oil bulk compared to the normal bulk. We know from previous experience that it is difficult to determine perfect bulks based on phenotypes for seed composition traits [18]. Thus, it is not surprising that our three populations did not all show similar enrichment. In fact, the A2 population did not show enrichment for this deletion in its high-oil bulk. It is worth noting, however, that the spread of the oil data in the A2 population was less distinct than the spread from the A1 and A3 populations (Figure 1), indicating that the A2 plants were more likely to have their DNA samples bulked into the wrong group. Thus, we tentatively conclude that the large deletion on chromosome 14 is likely co-segregating with the high-seed-oil phenotype in these mutant populations.

### 3.3. Functional Analysis

**Figure 3:**
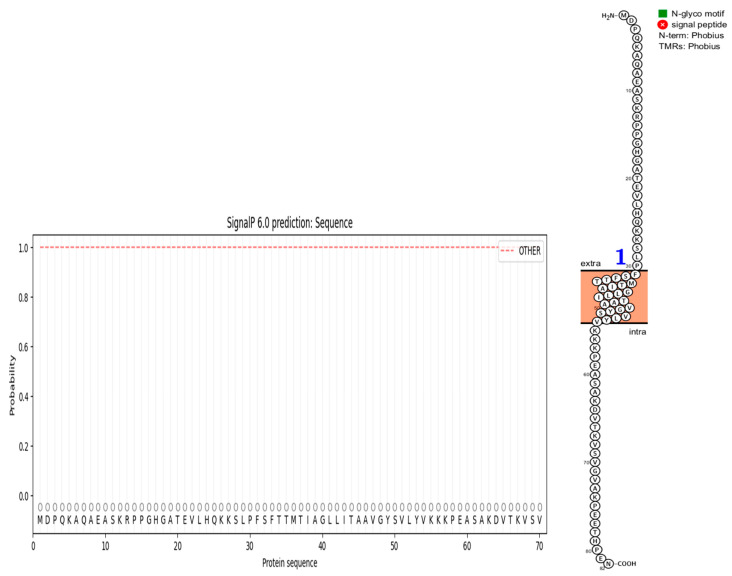
Predicted topology of *Glyma.14G101900*. It has one transmembrane region, designated by the blue 1. Extra means extra-cellular, and intra means inside the plasma membrane.

**Figure 4:**
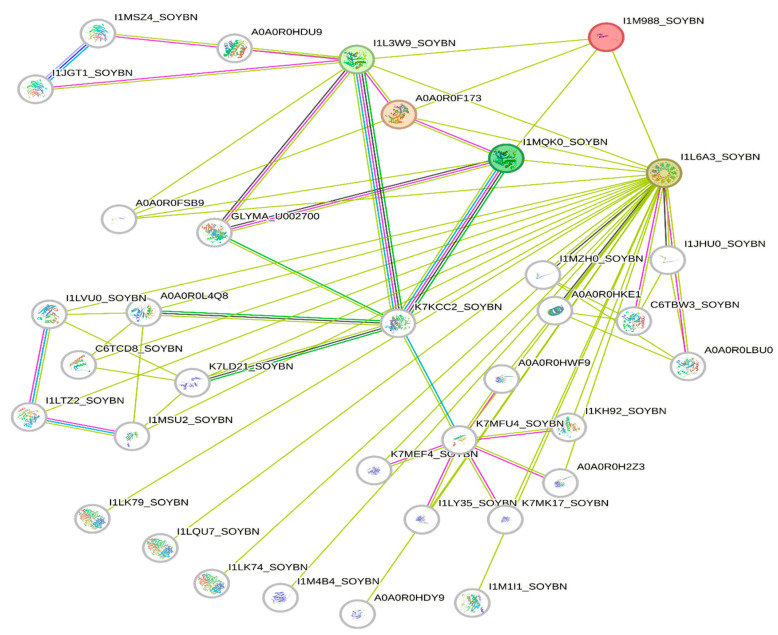
Protein network for *Glyma.14G101900* clustered in three groups. *Glyma.14G101900* is referred to as I1M988 (red color) in the STRING database. Green lines represent all relationships to *Glyma14G101900*, whereas other colors represent multiple unique connections between genes in the associated network families.

**Figure 5:**
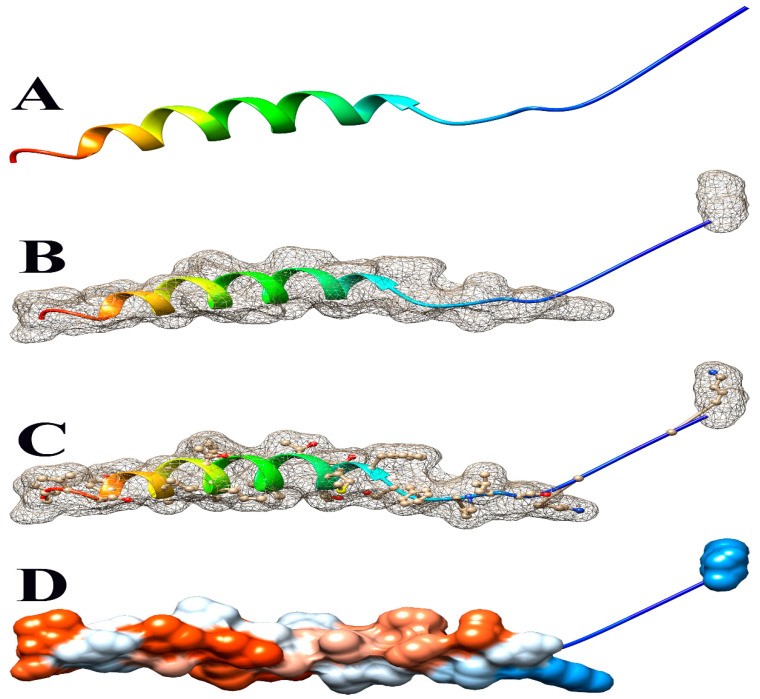
Some views of *Glyma.14G101900* PDB model predicted by Phyre2 and illustrated by Chimera. (**A**) Ribbon view, (**B**) mesh surface view, (**C**) mesh surface with ball and stick view, and (**D**) hydrophobicity surface (hydrophobicity surface preset from dodger blue for the most hydrophilic to white to orange-red for the most hydrophobic).

**Figure 6:**
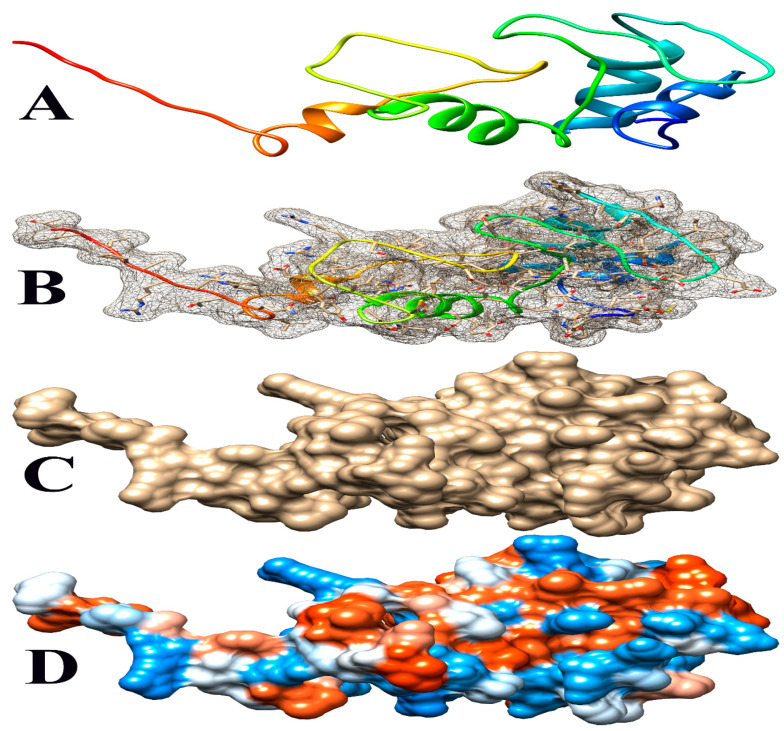
Some views of *Glyma.14G102100* PDB model predicted by Phyre2 and illustrated by Chimera. (**A**) Ribbon view, (**B**) mesh surface with ball and stick view, (**C**) surface view, and (**D**) hydrophobicity surface (hydrophobicity surface preset from dodger blue for the most hydrophilic to white to orange-red for the most hydrophobic).

The deletion on chromosome 14 encompasses 20 soybean gene models. All annotated functions of the 20 putative genes in the deleted region are summarized (Table 6). Of the 20 genes, 18 have a readily predicted function, but none appears to have an obvious connection to seed oil phenotypes. Table 6, Table 7 and Table 8 contain information about all genes within the deletion region. Among genes in this table, *Glyma.14G102100* and *Glyma.14G101900* had no readily predicted function. Therefore, bioinformatic analyses were performed to reveal possible functions for these genes. *Glyma.14G101900* with 82 amino acids is composed of 61% disordered protein (lacks an ordered three-dimensional structure) (Table 7) and is predicted to be a transmembrane protein by TAIR. The membrane topology of this gene was illustrated and predicted by Protter (Figure 3). The protein network for *Glyma.14G101900* (Figure 4) and the PDB model of both *Glyma.14G101900* and *Glyma.14G102100* were predicted by Phyre2 and illustrated by Chimera (Figure 5 and Figure 6).

## 4. Discussion

Forward screening of mutant populations for seed composition traits has been utilized with success in many crop species, including soybean seed composition changes induced by FN [18,21,22]. Soybean FN mutant families are powerful due to large variations in mutation sizes, from several bp deletions up to Mb-sized deletion events [17,18,19,24]. We utilized this resource and a forward screening approach to identify three mutants with high seed oil content and lower decreases in protein than are found in varieties produced using conventional breeding techniques [126,127,128]. These mutants were then backcrossed to the parent variety, which, in theory, should produce heterozygous offspring for the mutant traits in the F1 population. After a generation of self-pollination, we would expect to observe a genotypic segregation ratio of 1:2:1 for the homozygous mutant/heterozygous/homozygous wild type.

In the populations A1, A2, and A3, phenotype segregation was apparent in the F2:3 seeds, with oil content ranging from 19 to 24%, spread over a somewhat normal distribution. Assuming that the high-oil phenotype is caused by an FN-induced deletion, CGH may be able to show the deletion event when comparing the highest and lowest oil F2 bulked DNA samples, which was clearly observed in the A1 population. Furthermore, the size of the deletion detected, about 300 kb, is within the size range we anticipate and is frequently observed in CGH on soybean FN mutants [17,18,24].

While the size of the deletion event may imply that it is the source of observed phenotypic variation, it is also important to examine the deleted genes to begin to hypothesize about the mechanism which causes the observed variability. To date, many efforts have been made through mutagenesis, conventional breeding, and biotechnology to increase oil content of seeds. It has been well established that the ratio of sucrose/asn+gln from the mother plant significantly alters oil and protein content but results in an inverse correlation of these traits [129,130,131].

There is a genetic component to this, as high oil is heritable when selected in this manner, but usually results in a corresponding and roughly equal loss of protein. Metabolic engineering efforts have been effective at elevating oil content without the corresponding loss in protein by utilizing push, pull, and protect mechanisms. First, the “push” mechanism directs hydrocarbon resources toward the oil biosynthetic pathway, creating an abundant source of metabolic precursors. The transcription factor WRI1 is known to operate in this fashion [12,132,133]. Next, “pull” mechanisms occur later in the pathway and use downstream metabolites at a faster rate, thereby causing upstream resources to be re-directed into the pathway [11].

The enzyme diacylglycerol acyltransferase (DGAT) catalyzes the final and only dedicated step to TAG synthesis via the Kennedy pathway by combining a diacylglycerol molecule with an acyl-CoA [134]. Biochemical studies have also determined that this is a rate-limiting step in many species, so increasing the speed of TAG formation via DGAT increases the speed of the entire pathway [135]. DGAT-overexpression studies confirm this phenomenon, and DGAT-overexpressed plants have significantly higher oil levels, with no decrease in protein [136,137]. Lastly, “protect” mechanisms ensure that TAGs already formed do not degrade. For example, lipase knockouts [138] exhibit increased oil content, as do oleosin overexpressors, which are proteins that stabilize storage oil bodies [11]. Better yet, plants which are engineered with two or more of these steps exhibit synergistic increases in oil, such as tobacco leaves with up to 30% on a dry-weight basis of storage lipids [12].

In 1R22, the main FN mutant from this study, 20 gene models are located within a deletion that co-segregates with the high-oil phenotype. Eighteen of these genes have known functions but do not have an obvious fit to the high-seed-oil mutant phenotype. One of the unknown genes, *Glyma.14G102100*, is predicted to be a transposon ty3-g gag-pol polyprotein with 99.4% confidence and classified as a DNA-binding protein by Phyre2 (Table 7). We have no reason to suspect that this gene is involved in the mutant high-oil trait. The other unknown gene, *Glyma.14G101900*, has a COOH terminus predicted to be inside the plasma membrane, while the H2N terminal is predicted to be extra-cellular. A signal peptide analysis also showed that Glyma.14G101900 has no signal peptide (Figure 3). Some articles state that disordered proteins mainly trigger cellular stress responses or affect protein interaction networks. Ma et al. [139] stated that the deletion of such a disordered region enhances oil accumulation in Arabidopsis. The hydrophobicity surface and other views of the *Glyma.14G101900* and *Glyma.14G102100* PDB model predicted by Phyre2 and is illustrated via Chimera (Figure 5 and Figure 6).

Protein network analysis performed by the STRING database indicates that *Glyma.14G101900* has an interaction with four main proteins (STRING identifiers: I1L6A3, I1MQK0, A0A0R0F173, and I1L3W9), as shown in Figure 4. I1L3W9 is an uncharacterized protein that belongs to the short-chain dehydrogenase/reductase (SDR) family. A0A0R0F173 is AB hydrolase-1 domain-containing protein. I1MQK0 is also an uncharacterized protein that belongs to the short-chain dehydrogenases/reductases (SDR) family. SDR enzymes have critical roles in lipid metabolism [140].

I1L6A3 is a Seipin 1A that has a role in lipid droplet formation and storage, and it is necessary for both adipogenesis and lipid droplet (LD) organization [141]. At this stage, we do not know how a deletion of *Glyma.14G101900* would change the interactions between these proteins to produce high oil. It is possible that *Glyma.14G101900* has a negative interaction with these proteins, specifically Seipin, such that its deletion increases oil production.

We also speculate that one of the proteins in the deleted section may have a role in reducing triacylglycerol biosynthesis. Thus, many pathway analyses were performed, but no clear role of any of the gene products in triacylglycerol biosynthesis has been uncovered so far. The investigations of possible functions of these putative genes (especially from available RNA-seq data) are summarized in Table 8.

There seem to be several plausible hypotheses for how this deletion event may be influencing oil content in seeds; however, further studies are needed to confirm this. First, it would be useful to establish a genetic marker for this deletion to confirm that this location is the source of the high-oil phenotype. This could be a simple PCR amplicon across the deletion boundaries, which would provide a PCR product in plants that carry the deletion and no product in plants that do not carry the deletion. This would be analogous to a *VgDGAT* marker that was used to track a transgene conferring a high-oil phenotype [136,137]. In other CGH mutant lines, this method was used to confirm a FAD2 gene deletion, and the high oleic acid content correlated directly with the presence or absence of a PCR marker [17]. In addition, cloning of the genes in this region and inserting them into the mutant via transgenesis may be able to rescue the wild-type phenotype and precisely pinpoint the gene responsible for increased oil content. In the future, a full functional analysis could systematically reveal the candidate gene or genes within this deletion that underly the changes in phenotype. Ultimately, breeders and growers are not very concerned with the exact gene or mechanism which increases oil content; they know only that change is established as heritable, is easily identifiable with standard assays, and has limited other detrimental effects on the phenotype. Ultimately, it seems that the loss of a specific gene or genes within the deleted 300 kb region on chromosome 14 is responsible for the high-oil phenotype, and a further analysis could pinpoint the ultimate cause of these changes.

## Figures and Tables

**Table 1:** Mean oil content (on a dry weight basis) and the standard error in parentheses of three FN lines from the field in Minnesota (2013) and Lexington, KY (2015), and the parental line, determined on a dry-weight basis via bulk-seed NIRS. *n* = 3 plots in one location. Data are displayed as mean ± SE.

ID	Gen	MN Oil (2013)	KY Oil (2015)	Notes
1R22C28Cgadbr355aMN13	M8	22.0 ± 0.6	20.4 ± 1.3	High-oil, short, bushy, indeterminate, late maturity
5R12C21Dar387dMN13	M5	22.4 ± 0.8	20.1 ± 0.9	High-oil, erect petioles and lateral branches, slightly chlorotic small lanceolate leaves, late maturity
5R16C01Dar388eMN13	M5	21.9 ± 1.1	22.3 ± 1.2	High-oil, short, slightly chlorotic smaller lanceolate leaves, petioles long compared to plant ht., late maturity
M92-220	Parent	19.0 ± 0.7	19.4 ± 0.6	Parent of mutant lines

**Table 2:** Oil content of individual F1 seeds. Each F1 seed was assigned a population name for reference to the future generations raised from that seed. The first parent mentioned is the maternal plant, and the second mentioned is the paternal. Oil content was determined by single-seed NMR using a Minspec 20 (Bruker Biospin, The Woodlands, TX, USA). Seeds were weighed and placed into the tube and allowed to warm to 40 °C before insertion into the instrument. The standard oil seed measurement procedure supplied with the instrument controller was used. *n* = 3 technical replications on each seed, and SEs are in parentheses. Herein, the lines are referred to by the population name rather than parents for clarity.

Name	Parents	Mass (g)	OIL (% db)
A1	1R22×WT	0.2544	22.5 (0.19)
A2	1R22×WT	0.2181	22.8 (0.30)
A3	1R22×WT	0.2401	22.5 (0.26)
A4	5R12×WT	0.1742	19.3 (0.26)
A5	5R12×WT	0.2092	19.6 (0.33)
A6	5R12×WT	0.2027	21.6 (0.11)
A7	5R12×WT	0.2741	18.9 (0.27)
A8	WT×1R22	0.2734	19.3 (0.21)
B1	WT×1R22	0.1562	17.3 (0.37)
B2	WT×1R22	0.2771	18.9 (0.22)
WT	N/A	0.2051	20.5 (0.23)
1R22	N/A	0.1716	22.9 (0.36)
5R12	N/A	0.1706	20.5 (1.3)

**Table 3:** Protein and oil content of bulked F2 seeds. The “Cross” column shows the parents in the original cross; the first parent shown is the maternal plant, and the second mentioned is the paternal. Oil and protein content was determined by bulk-seed NIRS using a Perten DA7200 Spectrometer with n = 3 technical replications on each seed batch.

Plant ID	Cross	Mean Protein	SE	Mean Oil	SE
A1	1R22×WT	43.6	0.8	22.4	0.2
A2	1R22×WT	43.1	0.1	22.3	0.3
A3	1R22×WT	42.5	0.3	22.6	0.3
A4	5R12×WT	41.3	0.5	25.1	0.3
A7	5R12×WT	42.4	0.0	22.1	0.0
A8	WT×1R22	44.3	0.1	19.1	0.2
B1	WT×1R22	42.7	0.8	22.1	0.4
B2	WT×1R22	43.8	0.2	21.6	0.2
M92-220	Parent	42.6	0.2	22.5	0.2
5R12	Mutant	42.7	0.5	21.8	0.1
1R22	Mutant	38.6	0.5	24.6	0.4

**Table 4:** Single-seed oil and protein of 30 randomly selected F2 seeds (from F1 plants) selected for field planting, all of which are sibs. The field # (number) serves as an identifier related to seeds retained in long-term storage and used to track lineages. Oil and protein were determined for each seed using single-seed NIRS with 3 technical reps per seed. Single seeds were also surveyed in a similar way for lines A2, A3, A4, A8, B1, and B2, whose genetic lineages are described in Table 3.

Line	Field # (ID)	AVG Prot	SE Prot	AVG Oil	SE Oil
A1	1	45.8	0.5	20.9	0.2
A1	2	44.8	0.5	20.7	0.6
A1	3	46.1	0.6	21.1	0.2
A1	4	45.2	0.9	18.1	0.3
A1	5	45.1	0.6	20.7	0.1
A1	6	38.5	0.7	22.4	0.3
A1	7	42.6	0.4	22.2	0.5
A1	8	46.1	0.3	20.0	0.0
A1	9	41.2	0.3	22.3	0.4
A1	10	50.6	0.4	17.5	0.2
A1	11	47.3	0.3	19.6	0.1
A1	12	42.0	0.2	22.7	0.2
A1	13	38.6	1.0	22.4	0.6
A1	14	48.2	0.5	19.1	0.4
A1	15	44.0	1.2	21.0	0.5
A1	16	46.3	0.2	20.1	0.2
A1	17	53.1	0.9	17.3	0.6
A1	18	45.7	0.0	20.9	0.0
A1	19	48.3	0.2	19.7	0.7
A1	20	45.6	0.4	21.1	0.5
A1	21	45.5	0.4	19.8	0.4
A1	22	37.1	0.8	24.3	0.3
A1	23	46.1	0.3	19.6	0.4
A1	24	51.1	1.1	18.0	0.0
A1	25	46.9	0.4	19.7	0.2
A1	26	43.5	0.7	20.4	0.2
A1	27	43.3	0.3	23.7	0.5
A1	28	42.2	1.0	22.2	0.3
A1	29	49.8	1.0	19.0	0.4
A1	30	40.8	0.1	22.1	0.2

**Table 5:** Seed oil and protein from F2:3 lines A1, A2, and A3 (all originally derived from 1R22×WT crosses). Each row represents data from a sample of F3 seeds derived from an individual F2 plant. Oil and protein were determined via NIRS using a Perten DA7200 spectrometer and calculated on a 0% moisture basis. Each population (A1, A2, and A3) was split into a high-oil (bold text) and normal-oil (non-bold text) group. These groupings were used to determine which F2 DNA samples were bulked together for CGH analyses (high-oil bulk versus normal-oil bulk).

Plant ID	Mean Protein	Mean Oil
**MNA1: 37.5**	**40.1**	**24.2**
**MNA1: 11**	**39.3**	**24.2**
**MNA1: 14**	**40.2**	**24.1**
**MNA1: 16**	**39.0**	**24.0**
**MNA1: 44**	**38.6**	**24.0**
**MNA1: 33**	**39.0**	**23.3**
MNA1: 15	43.9	21.7
MNA1: 3	45.3	21.4
MNA1: 26	43.9	21.3
MNA1: 12	45.2	21.0
MNA1: 3	43.8	20.6
MNA1: 41.5	46.3	20.2
**MNA2: 8**	**40.1**	**24.1**
**MNA2: 28**	**39.9**	**23.9**
**MNA2: 29**	**39.6**	**23.8**
**MNA2: 40**	**38.9**	**23.8**
**MNA2: 4**	**39.2**	**23.7**
**MNA2: 43**	**39.8**	**23.7**
**MNA2: 31**	**38.6**	**23.4**
**MNA2: 5**	**39.5**	**23.4**
**MNA2: 25**	**40.2**	**23.2**
**MNA2: 33**	**39.8**	**23.2**
MNA2: 21	45.3	22.4
MNA2: 30	43.9	21.9
MNA2: 44.5	45.1	21.7
MNA2: 10	44.9	21.7
MNA2: 33.5	43.8	21.5
MNA2: 15	45.0	21.5
MNA2: 42	45.2	21.0
MNA2: 44	44.5	20.4
**MNA3: 7**	**39.5**	**24.3**
**MNA3: 42.5**	**39.0**	**24.0**
**MNA3: 3**	**40.2**	**23.6**
**MNA3: 8**	**40.9**	**23.5**
**MNA3: 14**	**39.1**	**23.4**
MNA3: 34	44.0	21.2
MNA3: 38.5	44.5	21.1
MNA3: 30.5	44.4	20.9
MNA3: 4	45.1	20.7
MNA3: 37.5	44.5	20.4
MNA3: 25	44.3	20.4
MNA3: 16	44.9	20.2
MNA3: 33	46.0	19.2
MNA3: 34.5	45.1	19.2

**Table 6:** A large deletion event was detected on Chr14, from position 9,994,086 to 10,301,954 (coordinates based on the Williams 82 genome version 2 assembly (Wm82.a2.v1), a span of approximately 308 kb in the 1R22-derived populations. * Putative transcription factors.

	Arabidopsis Homologue ID, NCBI Protein ID, KEGG ID	Chr: Gm14	Predicted Functions			
			Pfam, KEGG	GO, KOG, AT	PantherFam	UniProt
*Glyma.14G101000*	*AT5G10810.1,* NP_001241623, gmx:548093	Start: 10254421 Stop: 10258523, 128 orthologues 3 paralogues 7 domains and features, 45 oligo probes	Enhancer of rudimentary (PF01133), Glutaredoxin 2, C terminal domain (PF04399).	Positive regulation of Notch signaling pathway (GO:0045747), cell cycle (GO:0007049), pyrimidine nucleotide biosynthetic process (GO:0006221), enhancer of rudimentary (KOG1766)	Enhancer of rudimentary (PTHR12373)	Enhancer of rudimentary homolog (C6TKU9)
*Glyma.14G101100*	*AT5G10820.1,* XP_003545404, gmx:100806186	Start: 10268501 Stop: 10272945, 101 orthologues 12 paralogues. 23 domains and features, 49 oligo probes	Major Facilitator Superfamily (PF07690), The biopterin/folate transporter (PF03092)	Transmembrane transport (GO:0055085), integral component of membrane (GO:0016021)	Folate biopterin transporter 1, chloroplastic (PTHR31585)	Folate-biopterin transporter 6 (I1M978)
*Glyma.14G101200*	*AT4G31870.1,* *KAH1212471*	Start: 10274782 Stop: 10276509, 138 orthologues 13 paralogues. 12 domains and features, 11 oligo probes.	Glutathione peroxidase (PF00255)	Response to oxidative stress (GO:0006979), obsolete oxidation–reduction process (GO:0055114), Glutathione peroxidase activity (GO:0004602)	Glutathione peroxidase (PTHR11592)	Glutathione peroxidase (A0A0R0GIC5), Biological process: response to oxidative stress
*Glyma.14G101300*	*AT4G31870.1* *KAH1093928*	Start: 10277857 Stop: 10278815, 1 orthologue 7 domains and features, 20 oligo probes.	Glutathione peroxidase (PF00255)	Response to oxidative stress (GO:0006979), obsolete oxidation–reduction process (GO:0055114), Glutathione peroxidase activity (GO:0004602)	Glutathione peroxidase, (PF00255)	Glutathione peroxidase (A0A0R0GNK4), Biological process: response to oxidative stress
*Glyma.14G101400*	*AT4G31860.1,* *KAH1212475* gmx: K17499	Start: 10285154 Stop: 10291084, 63 orthologues 153 paralogues. 13 domains and features, 34 oligo probes	Protein phosphatase 2C (PF00481)	Protein dephosphorylation (GO:0006470), catalytic activity (GO:0003824), protein serine/threonine phosphatase activity (GO:0004722), serine/threonine protein phosphatase (KOG0699)	Protein phosphatase 2C (PTHR13832)	protein serine/threonine phosphatase (I1M981), ^Mg2+^ Mn^2+^ metal-ion binding, myosin phosphatase activity, Biological process: protein dephosphorylation
*Glyma.14G101500*	*AT4G31180.1,* *XP_003544559,* gmx:100788164	Start: 10297083 Stop: 10303442, 255 orthologues 14 paralogues 21 domains and features, 56 oligo probes.	tRNA synthetases class II (D, K and N) (PF00152), tRNA-synt_2d (PF01409), tRNA anti-codon (PF01336), KEGG: Mitochondrial biogenesis (K01876)	tRNA aminoacylation for protein translation (GO:0006418), nucleotide binding (GO:0000166), aminoacyl-tRNA ligase activity (GO:0004812), ATP binding (GO:0005524)	Aspartyl/lysyl-tRNA synthetase (PTHR22594)	Aspartate—tRNA ligase (I1M984), [ATP + L-aspartate + tRNA (Asp) = AMP + diphosphate + L-aspartyl-tRNA (Asp)] Cellular component: aminoacyl-tRNA synthetase multienzyme complex, cytosol Molecular function: aspartate-tRNA ligase activity, ATP binding, DNA binding, RNA binding Biological process: aspartyl-tRNA aminoacylation
*Glyma.14G101600*	*AT2G18193.1,* *KAH1212479.1*	Start: 10306650 Stop: 10310815, 492 orthologues 34 paralogues 8 domains and features, 6 oligo probes.	ATPase family associated with various cellular activities (AAA) (PF00004)	ATP binding (GO:0005524)	BCS1 AAA-type ATPase (PTHR23070)	ATPase AAA-type core domain-containing protein (A0A0R0GCB5), Molecular function: ATP binding, ATP hydrolysis activity
*Glyma.14G101700*	*AT5G25080.1* NP_001237643.1, gmx:100500667	Start: 10314547 Stop: 10319982, 13 orthologues. Five domains and features, 53 oligo probes	Sas10/Utp3/C1D family (PF04000) KEGG: Eukaryotic RNA degradation (100500667), Messenger RNA biogenesis (K12592)	KOG: DNA-binding protein C1D involved in regulation of double-strand break repair (KOG4835),AT: Sas10/Utp3/C1D family (*AT5G25080.1*)	Sun-cor steroid hormone receptor co-repressor, (PTHR15341), Nuclear nucleic acid-binding protein (PTHR15341:S3)	Nuclear nucleic acid-binding protein C1D (C6T2F3). Plays a role in the recruitment of the exosome to pre-rRNA to mediate the 3′-5′ end processing of the 5.8S rRNA. Cellular component: cytoplasm, exosome, nucleolus Molecular function: DNA binding, RNA binding Biological process: maturation of 5.8S rRNA, regulation of gene expression
*Glyma.14G101800*	*AT4G31840.1,* *KAG4962690.1*	Start: 10327216 Stop: 10328729, 57 orthologues and 5 paralogues 14 domains and features, 40 oligo probes	Plastocyanin-like domain (PF02298)	Electron transfer activity (GO:0009055) AT: Early nodulin-like protein 15 (*AT4G31840.1*)	Blue copper protein JGI N/A IEA (PTHR33021),	Phytocyanin domain-containing protein (I1M987) Cellular component: plasma membrane. Molecular function: electron transfer activity
*Glyma.14G101900*	*AT4G31830.1,* *KAG4962691.1*	Start: 10329690 Stop: 10330667, 101 orthologues and 1 paralogue 5 domains and features, 26 oligo probes		AT: Transmembrane protein (*AT4G31830.1*)	OS09G0127700 PROTEIN (PTHR33919)	Transmembrane protein (I1M988). Cellular component: membrane
*Glyma.14G102000*	*AT5G10840.1*, XP_003544560.1, gmx:100788693	Start: 10332624 Stop: 10337739, 286 orthologues and 28 paralogues. Twenty domains and features, 34 oligo probes	Endomembrane protein 70 (PF02990), Major Facilitator Superfamily (PF07690) KEGG: Exosome (K17086)	Integral component of membrane (GO:0016021) AT: Endomembrane protein 70 protein family (*AT5G10840.1*)	Transmembrane 9 superfamily protein (PTHR10766)	Transmembrane 9 superfamily member (I1M989) Cellular component: endosome membrane, Golgi membrane, membrane Biological process: protein localization to membrane
*Glyma.14G102100*	None *KAH1093938.1*	Start: 10093844 Stop: 10094237, 34 paralogues. One domain and feature				Uncharacterized protein (A0A0R0GBJ6)
*Glyma.14G102200* *	*AT1G18400.1,* *KAH1093939.1*	Start: 10350687 Stop: 10352402, 119 orthologues and 79 paralogues. Ten domains and features, 9 oligo probes		AT: Encodes the brassinosteroid signaling component *BEE1* (*BR-ENHANCED EXPRESSION 1*). Positively modulates the shade avoidance syndrome in Arabidopsis seedlings. (*AT1G18400.1*)	Sterol regulatory element-binding protein (PTHR12565) Transcription factor bee 3 1 hit (PTHR12565:SF340)	BHLH domain-containing protein (A0A0R0GBI9), Cellular component: nucleus Molecular function: DNA-binding transcription factor activity, protein dimerization activity
*Glyma.14G102300*	*AT1G21280.1*	Start: 10115205 Stop: 10116250, 1 orthologue and 4 paralogues. Four domains and features	Retrotran_gag_2 1 hit (PF14223)	AT: Copia-like polyprotein/retrotransposon (*AT1G21280.1*)	Retrotran_gag_3 domain-containing protein 1 hit (PTHR47481:SF19)	Retrotran_gag_3 domain-containing protein (A0A0R0GBC5)
*Glyma.14G102400*	*AT1G19260.1* XP_014622176.2 gmx:102660685	Start: 10409336 Stop: 10412210, 53 orthologues and 83 paralogues. Seven domains and features.	Domain of unknown function DUF4371 (PF14291) hAT family C-terminal dimerisation region (PF05699) KEEG: LOW QUALITY PROTEIN: zinc finger MYM-type protein 1 (102660685)	AT: Encodes a ceramide synthase that uses very long chain fatty acyl-CoA and trihydroxy LCB substrates (*AT1G19260.1*)	General transcription factor 2-related zinc finger protein (PTHR11697) Zinc finger, mym domain-containing 1 1 hit (PTHR11697:SF227)	TTF-type domain-containing protein (K7M603)
*Glyma.14G102500*	*AT4G31820.1*, XP_003544563.1, gmx:100790291	Start: 10424224 Stop: 10429543, 159 orthologues and 75 paralogues, 18 domains and features and maps to 29 oligo probes.	BTB/POZ domain (PF00651) NPH3 family (PF03000) KEGG: BTB/POZ domain-containing protein NPY1 (100790291)	Animal organ development (GO:0048513) auxin transport (GO:0060918) obsolete signal transducer activity (GO:0004871) protein binding (GO:0005515) AT: A member of the *NPY* family genes (*NPY1*/*AT4G31820*, *NPY2/AT2G14820, NPY3/AT5G67440, NPY4/AT2G23050, NPY5/AT4G37590*). Encodes a protein with similarity to *NHP3*. Contains BTB/POZ domain. Promoter region has canonical auxin response element-binding site and Wus-binding site. Co-localizes to the late endosome with PID. Regulates cotyledon development through control of *PIN1* polarity in concert with PID. Also involved in sepal and gynoecia development. *AT4G31820.1*	OS12G0117600 Protein (PTHR32370) BTB/POZ Domain-containing protein *NPY1* (PTHR32370:SF7)	*NPH3* domain-containing protein (K7M604), Pathway: Protein modification, protein ubiquitination Biological process: protein ubiquitination
*Glyma.14G102600*	*AT4G31810.1*, XP_028198380.1	Start: 10431488 Stop: 10441267 117 orthologues and 19 paralogues. Eight domains and features, 31 oligo probes	Enoyl-CoA hydratase/isomerase (PF00378), ECH_2 1 hit (PF16113) KEGG: beta-Alanine metabolism (K05605), Valine, leucine and isoleucine degradation (map00280), beta-Alanine metabolism (map00410), Propanoate metabolism (map00640), Metabolic pathways (map01100), Carbon metabolism (map01200)	Metabolic process (GO:0008152), Catalytic activity (GO:0003824) AT: ATP-dependent caseinolytic (Clp) protease/crotonase family protein (AT4G31810.1)	Enoyl-coa hydratase-related (PTHR11941)	3-hydroxyisobutyryl-CoA hydrolase (I1M992) Hydrolyzes 3-hydroxyisobutyryl-CoA (HIBYL-CoA), a saline catabolite. Has high activity toward isobutyryl-CoA. Could be an isobutyryl-CoA dehydrogenase that functions in valine catabolism. Catalytic activity 3-hydroxy-2-methylpropanoyl-CoA + H_2_O = 3-hydroxy-2-methylpropanoate + CoA + H^+^, Molecular function 3-hydroxyisobutyryl-CoA hydrolase activity. Biological process: valine catabolic process, mitochondrial
*Glyma.14G102700*	*AT5G10870.1*, XP_006596036.1	Start: 10445611 Stop: 10448775 174 orthologues and 9 paralogues, 9 domains and features 19 oligo probes.	KEGG: Phenylalanine, tyrosine and tryptophan biosynthesis (K01850), Phenylalanine, tyrosine and tryptophan biosynthesis (map00400), metabolic pathways (map01100), Biosynthesis of secondary metabolites (map01110), biosynthesis of amino acids (map01230)	Aromatic amino acid family biosynthetic process (GO:0009073), Chorismate mutase activity (GO:0004106), Chorismate mutase (KOG0795) AT: Encodes chorismate mutase AtCM2 (AT5G10870.1)	Chorismate mutase (PTHR21145)	Chorismate mutase (A0A0R0GIU8), Cellular component: cytoplasm, Molecular function: Chorismate mutase activity. Biological process: amino acid biosynthetic process, aromatic amino acid family biosynthetic process, chorismate metabolic process
*Glyma.14G102800*	*AT2G25050.1*, KAG5110165.1	Start: 10486626 Stop: 10508579, 240 orthologues and 36 paralogues, 57 domains and features, 28 oligo probes.	C2 domain of PTEN tumor-suppressor protein (PF10409), DUF4283 1 (PF14111), *FH2 2* (PF02181), PTEN_C2 1 (PF10409), RVT_1 1 (PF00078)	AT: Class II formin; modulator of pollen tube elongation (*AT5G58160.1*)	Formin-related (PTHR23213), FORMIN-J 1 (PTHR45733) FORMIN-J 1 (PTHR45733)	Formin-like protein A0A368UH40 Cellular component: membrane Molecular function: phosphoprotein phosphatase activity
*Glyma.14G102900* *	*AT1G80840.1*, XP_014622429.1, gmx:100791870	Start: 10511024 Stop: 10513622 80 orthologues and 44 paralogues. 10 domains and features, 47 oligo probes.	*WRKY* DNA-binding domain (PF03106), Takusan (PF04822) NUDE_C (PF04880)	Regulation of transcription, DNA-templated (GO:0006355), DNA-binding transcription factor activity (GO:0003700), Sequence-specific DNA binding (GO:0043565), AT: *WRKY DNA-BINDING PROTEIN 40* (*AT1G80840.1*) Pathogen-induced transcription factor	*WRKY* transcription factor 36-related (PTHR31429), *WRKY* transcription factor 40-related 1 (PTHR31429:SF38)	*WRKY* domain-containing protein (I1M995), Cellular component: nucleus Molecular function: DNA-binding transcription factor activity, sequence-specific DNA binding

**Table 7:** Phyre2 results for unknown genes (Glyma.14G101900 and Glyma.14G102100).

Gene ID	Protein Seq	Confidence and Coverage PDB Molecule			PDB Header	Details
*Glyma.14G101900*	MDPQKAQAEASKRPPGHGATEVLHQKKSLPFSFTTMTIAGLLITAAVGYSVLYVKKKPEASAKDVTKVSVGVAKPEETHPEN	82	Confidence: 5.2% Coverage: 33%	na(+)/h(+) antiporter subunit b	Structure of bacillus pseudofirmus Mrp antiporter complex, monomer	61% of this sequence is predicted to be disordered. Disordered-region structures cannot be meaningfully predicted.
*Glyma.14G102100*	MVGEEEEPDWMTPYKNFLTQGVLPSHDNEVRCLKWKANYYIILDGELLKRGLIASLLKCLNNQQTDYVIRELHEGICALYIGGRSLATKVTLLTLQRDVDDARSLQTFRAPLLTISIV	118	Confidence: 99.4% Coverage: 88%	Transposon ty3-g gag-pol polyprotein	DNA-binding protein	

**Table 8:** Review of putative function of 19 Arabidopsis homologues of the genes in the deleted region of the high-oil soybean mutant.

Name of Gene	Function Reported	References
*AT5G10810*	As one of 25 candidate *AtPNP*-As which showed weak interaction strength in the yeast two-hybrid (Y2H) analysis. *AtPNP*-A = plant natriuretic peptides (PNPs), which comprise a novel class of hormones that systemically affect salt and water balance and responses to plant pathogens.	[34]
*AT5G10820*	A Folate–Biopterin Transporter (*FBT*) family member. *FBT*s are essential cofactors in one-carbon metabolism. The *FBT* family belongs to the major facilitator superfamily (*MFS*) and contains 12 transmembrane α-helices.	[35]
*AT4G31870*	*Glutathione peroxidases 7*(*GPX7*) is one of the major ROS-scavenging enzymes which catalyze the reduction of H_2_O_2_ in order to prevent potential H_2_O_2_-induced cellular damage. *GPX7* (Cys-108, Gln-143, and Trp-197) residues are potential catalytic residues found to be strictly conserved.	[36]
	*GPX7* is linked to the establishment of the photooxidative stress tolerance and the basal resistance to *P*. *syringae* infection.	[37]
	*GPX7* belongs to a family of thiol-based glutathione peroxidases that catalyzes the reduction of H_2_O_2_ and hydroperoxides to H_2_O or alcohols using glutathione as an electron donor. Plant GPXs are implicated in redox signal transduction.	[38]
	*VaAQ* (a putative GARP-type transcription factor of Amur grape (*Vitis amurensis*) overexpression increases antioxidant enzyme activities and upregulates ROS scavenging-related genes such as *GPX7* under cold stress.	[39]
	Strongly induced in carotenoid-accumulating Arabidopsis roots.	[40]
	Expressed highly in response to oxidation–reduction processes	[41]
	Molecular analysis indicates that *glutathione peroxidase 7* (*GPX7*) is specifically induced to compensate for the absence of APx-R (ascorbate peroxidase-related). (Peroxidases are enzymes that catalyze the reduction of hydrogen peroxide, thus minimizing cell injury and modulating signaling pathways in response to this reactive oxygen species.)	[42]
	The transcript abundance of the *GPX7* (*At4g31870*) was increased by a cryoprotectant treatment.	[43]
	*GPX7* (*At4g31870*) is increased upon auxin application.	[44]
*AT4G31860*	A protein phosphatase DEGs involved in Cold Response.	[45]
	AP2C18, highly ABA-inducible.	[46,47]
	Potentially involved in sucrose-induced atrazine tolerance. Protein phosphatase 2C, putative/PP2C, putative.	[48]
	One of the differentially expressed genes related to plant hormone signal transduction pathways. Putative function: abscisic acid (aba) signal transduction.	[49]
	ABA-induced genes in guard cells of Arabidopsis.	[50]
*AT4G31180*	Mutations in *At4g31180* cause the ibi1 (induced disease immunity) phenotype and can block BABA-IR in the background of SA-producing Col-0. (β-aminobutyric acid (BABA) is a priming agent that provides broad-spectrum disease protection.) An aspartyl tRNA synthase (AspRS) orthologue (*At4g31180*) that improves tolerance to biotic stress. The *At4g31180* is a target of a synthetic isomer of GABA, called BABA (β-Amino Butyric Acid). Aspartyl tRNA synthetase (AspRS) IBI1 in Arabidopsis thaliana (Arabidopsis) acts as an enantiomer-specific receptor of BABA. The primary function of AspRS enzymes is the charging of tRNAAsp with L-aspartic acid (L-Asp) for protein biosynthesis.	[51]
	One of the genes that is sensitive to infection through the NPR1- or JAR1-dependent pathways.	[52]
	*AT4G31180* (IBI1: impaired in BABA-Induced Disease Immunity 1) is one of the proteins identified in the seed monosome and polysome fractions that, based on their annotation, have been associated with RNA binding.	[53]
	The *IBI1* Receptor of β-Aminobutyric Acid interacts with VOZ transcription factors to regulate abscisic acid signaling and callose-associated defense.	[54]
	*AT4G31180* is a putative interaction partner of *AtGRXS17* (Arabidopsis Glutaredoxin S17).	[55]
	One of the fifty-three predicted genes with similarities to aminoacyl-tRNA synthetases identified in A. thaliana.	[56]
*AT2G18193*	*AT2G18193* is a P-loop nucleoside triphosphate hydrolases that only displayed significant induction upon X-irradiation in wild-type seedlings and not in imbibed seeds or sog1 mutants. (The transcription factor *SUPPRESSOR OF GAMMA 1* (*SOG1*), which is unique to plants but functionally similar to the mammalian tumor suppressor p53.)	[57]
	Expression level of (AAA-ATPase At2g18193-like, P-loop containing nucleoside triphosphate hydrolases superfamily protein) genes that are elevated in MSC (Transcriptome Analyses of Mosaic) mitochondrial mutants of cucumber lines. (DNA repair mechanisms are the regulation of proteolytic processes.)	[58]
	A gene regulated by sulfur deficiency encoding a protein with possible ATPase activity and metal-ion binding.	[59]
	A gene whose expression is ABA-inducible in the wild type of Arabidopsis but not in the ros1-4 (*REPRESSOR OF SILENCING 1* (*ROS1*)) mutant.	[60]
	A gene that is significantly upregulated in *pTSPO-PDC1* under drought stress compared to WT plants. (TSPO: tryptophan-rich sensory protein.)	[61]
	One of the 50 genes upregulated by TBM treatment. (Acetolactate synthase (ALS)-inhibiting herbicide tribenuron methyl (TBM).)	[62]
*AT5G25080*	Encodes a protein with high homology to Rrp47p and is encoded by At5g25080. (Rrp47p is an exosome-associated protein required for the 3′ processing of stable RNAs, Mitchell et al., 2003.)	[63]
	Candidate gene that includes significantly associated SNPs for traits involved in drought tolerance. Sas10/Utp3/C1D family.	[64]
	*AT5G25080 (RRP47*) is a known RNA exosome component with the RRP6 cofactor in plants that mediates protein–protein interactions.	[65]
*AT4G31840*	Oxido-reductases family: blue copper-binding proteins. A CWP or CW transcript differentially accumulated at a given growth temperature in floral stems.	[66]
	Highly repressed under *FPS* and *SQS* expression on the transcription of nuclear genes. (Squalene biosynthesis genes *FARNESYL DIPHOSPHATE SYNTHASE* (*FPS*) and *SQUALENE SYNTHASE* (*SQS*) were engineered via the *Nicotiana tabacum.*	[67]
	Early nodulin-like protein 15, increased abundance in HS (Humic Substances) treated vs. untreated roots of Arabidopsis.	[68]
	*AT4G31840* (*ENODL15*). Protein–protein interaction networks linked to aliphatic and indole glucosinolate biosynthetic pathways in Arabidopsis.	[69]
	One of the top 30 genes that are most downregulated in myb3r1 myb3r4 seedlings. (Mutations in *MYB3R1* and *MYB3R4* cause pleiotropic developmental defects and preferential downregulation of multiple G2/M-specific genes in Arabidopsis.)	[70]
*AT4G31830*	A gene that may be involved in the drought response and upregulated in leaf tissue.	[64]
	A gene upregulated in the NAE (*N*-Acylethanolamines) (and ABA-seedling arrays) annotated as “embryo associated” (e.g., late embryogenesis abundant genes, dehydrins, globulins, oleosins, and vicilins).	[71]
	A conserved drought-adaptive gene whose function is unclassified.	[72]
	A gene that shows tissue specificity, as well as expression conservation in rice and Arabidopsis seeds.	[73]
	Homologue to (Mtr.17894.1.S1_at) a putative ABI3 regulon of *Medicago truncatula.*	[74]
*AT5G10840*	Endomembrane protein (70 protein family) that is downregulated by *Colletotrichum acutatum* in strawberry crown tissue.	[75]
	AT5G10840 encodes a highly altered redox-regulated protein in response to 3 mM bicarbonate treatment in *A. thalina* var. *Landsberg erecta*.	[76]
	A conserved syntenic region that pairs between the pseudo-ancestral *Arabidopsis* genome and *Prunus* genetic maps. Endomembrane protein 70, putative TM4 family.	[77]
	The transmembrane proteins identified from the plasma membrane of poplar differentiating xylem and phloem. Endomembrane protein 70, putative.	[78]
*AT1G18400*	*BRASSINOSTEROID ENHANCED EXPRESSION1* (*BEE1*) (*At1g18400*) is a low-temperature regulator of flavonoid accumulation. *BEE1* and *GFR* (*G2-LIKE FLAVONOID REGULATOR*) were both shown to negatively regulate anthocyanin accumulation by inhibiting anthocyanin synthesis genes via the suppression of the bHLH (*TRANSPARENT TESTA8* (*TT8*) and *GLABROUS3* (*GL3*)) and/or the MYB (*PRODUCTION OF ANTHOCYANIN PIGMENTS2* (*PAP2*)) components of the MBW complex.	[79]
	Arabidopsis *BEE1* (*AT1G18400*) is the orthologue of bHLH056 in papaya. bHLH056 may be involved in the process of ABA stress but has different function compared to Arabidopsis.	[80]
	A positive regulator of flavonoid accumulation at low temperatures.	[79]
	A brassinosteroid signaling component and a positive regulator of shade avoidance syndrome.	[81]
	The putative *BEE1* (c42857_g1_i1_AT1G18400) showed lower expression in ovules at 16 DAA (days after anthesis) in small-seeded litchi.	[82]
	A gene encoding the BR (brassinosteroid) signaling components *EE3* (*AT1G73830*) and *EE1* (*AT1G18400*) that are significantly upregulated by ethanol treatment, suggesting that the BR pathway is also involved in plant responses to ethanol.	[83]
	*BEE1*, BR-related transcription factor that are upregulated by BR which encode putative *AtMYC2* (bHLH) proteins in A. thaliana.	
	One of the three redundant brassinosteroid early response genes that encode putative bHLH transcription factors required for normal growth.	[84]
	One of differential expression genes related to flowering in the Photoperiod pathway.	[85]
	*BEE1* is a positive regulator of photoperiod flowering and promotes flowering by directly binding to the floral integrator FT.	[86]
	*BEE1, -2*, and *-3* are negative regulators of photomorphogenesis.	[87]
	Involved in the response to iron deficiency.	[88]
*AT1G21280*	A SNP predicted to be associated with brown rot resistance in peach.	[89]
	Homologue to Tp57577_TGAC_v2_mRNA41271.v2 gene transcription involved in regrowth influenced by location and environmental conditions response after mowing of red clover (*Trifolium pratense*).	[90]
	Is a duplicated region in Chr 10 of soybean associated with seed protein content.	[91]
*AT1G19260*	*LOH3* (*At1g19260*)-encoded ceramide synthases use very long chain fatty acyl-CoA and trihydroxy long-chain base) LCB (substrates. Overexpression of *LOH1* and *LOH3* resulted in a significant increase in plant size. *LOH1* and *LOH3* overexpression results in a significant increase in cell number of root meristems. In contrast to results from *LOH2* overexpression lines, *LOH1* and *LOH3* overexpression results in little change in total sphingolipid content and composition of plants relative to wild-type controls, although small but significant reductions in C16 fatty acid-containing sphingolipids were detected as a result of minor changes throughout the sphingolipidome.	[92]
	Is a zinc finger protein-coding gene. Transposes transcription factor-type zinc finger protein with a HAT dimerization domain.	[93]
*AT4G31820*	Upregulation related to either auxin metabolism, transport, signaling, or response.	[94]
	Predicted target genes of *Gnetum gnemon* miRNAs against *Arabidopsis thaliana.*	[95]
	One of the shade-regulated *KAN1* target genes	[96]
	Functions redundantly in auxin-mediated organogenesis and root gravitropism with the *AGC3* (protein kinase A, cGMP-dependent protein kinase, and protein kinase C) kinase family	[97]
	*AT4G31820* (*ENP1/NPY1)* is in the *PGP* Family of Auxin Transport Facilitators. Role in regulation of Auxin Pathway Genes by *REV* and *KAN*. (*KANADI1* (*KAN1*), a member of the GARP family of transcription factors, a key regulator of abaxial identity, leaf growth, and meristem formation in *Arabidopsis thaliana*., *REVOLUTA* (*REV*).)	[98]
	All five *NPY* genes (*NPY1* = *At4g31820*, *NPY2* = *At2g14820*, *NPY3* = *At5g67440*, *NPY4* = *At2g23050*, and *NPY5* = *At4g37590*) were expressed in tips of *Arabidopsis* primary roots, but they displayed unique and overlapping patterns. NPY genes play an essential role in root gravitropic responses in Arabidopsis.	[99]
	*NPY* genes and *AGC* kinases define 2 key steps in a pathway that controls *YUC*-mediated organogenesis in *Arabidopsis*.	[100]
	Plays a critical role in auxin-regulated organogenesis in *Arabidopsis.*	[101]
*AT4G31810*	(*CHY4*, *At4g31810*) is a putative mitochondrial enzyme in valine degradation. A null mutant of 3-hydroxyisobutyryl-CoA hydrolase (*CHY4*, *At4g31810*) resulting in an embryo lethal phenotype. *CHY4* is essential for embryo development.	[102]
	*CHY4* involved in leucine degradation and exhibited a strong association with leucine levels in dark-related datasets.	[103]
	Candidate gene tagged by the associated SNPs related to four important fatty acid (erucic acid, oleic acid, linoleic acid, and linolenic acid) biosynthesis and metabolism in Brassica napus. Homologue to *BnaA08g12350D*. Enoyl-CoA hydratase/isomerase family protein.	[104]
	Downregulated as substrates of the *AtICP55 protein*. *AtICP55* is a secondary processing mitochondrial peptidase.	[105]
	Identified as one of conserved syntenic regions pairs between the pseudo-ancestral *Arabidopsis* genome and *Prunus* genetic maps. Function: enoyl-CoA hydratase/isomerase family protein.	[77]
	Candidate stigma-specific gene from S. squalidus. Function: Cys protease	[106]
	Involved in Leu degradation. Function: enoyl-CoA hydratases.	[107]
*AT5G10870*	Chorismate mutase gene involved in VTE (vitamin E) biosynthesis. Chloroplastic.	[108]
	Upregulated gene in the resistant genotype (*Myzus persicae*) after GPA infestation. Shikimate pathway, chorismate mutase 2	[109]
	*AthCM2* (*AT5G10870*) involved in the shikimate pathway that directs bulk carbon flow toward biosynthesis of aromatic amino acids.	[110]
	Contributes to phenylalanine biosynthesis in Arabidopsis.	[111]
	Involved in shikimate and phenylalanine biosynthesis in plants and algae.	[112]
	The activity of *AtCM2* appears to be insensitive to Phe and Tyr. (The first committed step of Phe biosynthesis from chorismate is catalyzed by chorismate mutase (*CM*).)	[113]
	Involved in metabolic pathways of amino acids and their associated genes.	[114]
	Belonging to the Asp family and the aromatic amino acid (AAA) networks.	[115]
*AT2G25050*	Encoding the actin-binding formin homology *FH2* protein. Around one-third of these *CHG-DMCs* (cytosine methylation sequence) located within a 3.3 kb region on chromosome 2 within the gene *At2g25050*.	[116]
	*AT2G25050* (*AtFH18*) is 81% close phylogenetic relationships to *GmFH3* in *G. max.*	[117]
	Associated with the cell cycle classification and involves the division of specific cells to form the final apple fruit shape.	[118]
*AT1G80840*	*AT1G80840* (*WRKY40*) encodes a pathogen-induced TF and harbors five associated distal peaks with its promoter.	[119]
	A conserved double-W box in the promoter of *CaWRKY40* mediates autoregulation during response to pathogen attack and heat stress in pepper.	[120]
	Associated with plant defense response.	[121]
	Codes for a pathogen-induced transcription factor.	[122]
	Common downstream gene of SA and upregulation of this gene diminished at high temperature by SA.	[123]
	Overexpression of *AtWRKY40* enhanced drought stress responses, presumably by interfering with the reactive oxygen species (ROS)-scavenging pathway and osmolyte accumulation process.	[124]
	Involved in the response to abiotic stresses.	[125]

## Data Availability

All relevant and pertinent data are made available in the manuscript. We encourage anyone with questions to contact William Serson for further information: wrs5272@psu.edu.
